# Knowledge of reproductive and sexual rights among University students in Ethiopia: institution-based cross-sectional

**DOI:** 10.1186/1472-698X-13-12

**Published:** 2013-02-13

**Authors:** Yohannes Mehretie Adinew, Abebaw Gebeyehu Worku, Zelalem Birhanu Mengesha

**Affiliations:** 1 Department of Reproductive Health, Wolaita Sodo University, Wolaita Sodo, Ethiopia; 2 Department of Reproductive Health, Institute of Public Health, University of Gondar, Gondar, Ethiopia

**Keywords:** Reproductive Health, Reproductive and Sexual Rights, Youths, Ethiopia

## Abstract

**Background:**

People have the right to make choices regarding their own sexuality, as far as they respect the rights of others. The knowledge of those rights is critical to youth’s ability to protect themselves from unwanted reproductive outcomes. Reproductive health targeted Millennium Development Goals will not be achieved without improving access to reproductive health. This study was aimed to assess knowledge of reproductive and sexual rights as well as associated factors among Wolaita Sodo University students.

**Methods:**

An institution-based cross-sectional survey was conducted among 642 regular undergraduate Wolaita Sodo University students selected by simple random sampling. A pretested and structured self-administered questionnaire was used for data collection. Data were entered using EPI info version 3.5.3 statistical software and analyzed using SPSS version 20 statistical package. Descriptive statistics was used to describe the study population in relation to relevant variables. Bivariate and multivariate logistic regression was also carried out to see the effect of each independent variable on the dependent variable.

**Results:**

More than half (54.5%) of the respondents were found to be knowledgeable about reproductive and sexual rights. Attending elementary and high school in private schools [AOR: 2.08, 95% CI: 1.08, 3.99], coming from urban areas [AOR: 1.46, 95% CI: 1.00, 2.12], being student of faculty of health sciences [AOR: 2.98, 95% CI: 1.22, 7.30], participation in reproductive health clubs [AOR: 3.11, 95% CI: 2.08, 4.65], utilization of reproductive health services [AOR: 2.34, 95% CI: 1.49, 3.69] and discussing sexual issues with someone else [AOR: 2.31, 95% CI: 1.48, 3.62], were positively associated with knowledge of reproductive and sexual rights.

**Conclusion:**

The level of knowledge of students about reproductive and sexual rights was found to be low. The Ministry of Education has to incorporate reproductive and sexual rights in the curricula of high schools and institutions of higher learning.

## Background

Reproductive and sexual health rights are rights of all people, regardless of age, gender and other characteristics. That is, people have the right to make choices regarding their own sexuality and reproduction, provided that they respect the right of others. Reproductive and sexual rights were first officially recognized at the International Conference on Population and Development (ICPD) in Cairo in 1994 [1].

The Program of Action of ICPD recognized that meeting the reproductive health (RH) needs is a vital requirement for human and social development. Protecting and promoting the reproductive and sexual rights of the youth and empowering them to make informed choices is a key to their wellbeing [2,3]. Action for universal access to sexual and reproductive health (SRH) by 2015 was agreed by 179 countries; despite this international commitment, progress towards the program has been slow [4].

Reproductive health targeted Millennium Development Goals (MDGs) will not be achieved without improving access to RH [5]. The majority of the young people have very little knowledge on what sexual rights they are entitled to. Sometimes, they do not even appreciate the extent of their violations and worse still they do not know where they could go, for legal or social advice [6,7]. Sources of information the youth traditionally access, such as parents have a skewed conception on anything related to sexuality and approach it from a cautionary perspective rather than from an informative one. Formal education on sexuality is limited to RH which is commonly found in the science syllabi and biology and does not cover reproductive and sexual rights [8].

The youth are unable to deal with such violations because of barriers like shame, guilt, embarrassment, not wanting friends and family to know; confidentiality; and fear of not being believed [9] and partly because of their inadequate knowledge and experience on sexuality issues including legal instruments that may accord them an opportunity to claim and protect sexuality-related rights [8].

Young people face increasing pressures regarding sex and sexuality including conflicting messages and norms which may be perpetuated by lack of awareness of their rights and results in many young people being either unable to seek help when they need it, and may prevent them from giving input within policy and decision making processes [10].

Evidences from Ethiopian Universities revealed that violations are rampant and inadequately addressed [11]. The in and out of school Ethiopian youth in the age range of 15 and 24 years face lots of challenges and constitute the highest percentage of new HIV cases in the country [12]. Traditional practices such as early marriage, marriage by abduction, and female genital cutting adversely affect the health and wellbeing of young people [13].

Therefore, this study set out to assess the level of youth knowledge about reproductive and sexuality rights and thereby generate appropriate intervention strategies so that mainstream issues of sexuality and related rights may be included in policies and university curricula.

## Methods

An institution-based, cross-sectional study was conducted among Wolaita Sodo University students at Sodo town which is located in Wolaita Zone of Southern Nations Nationalities and People’s Regional State (SNNPR). Sodo is 327 km from Addis Ababa. The university had 7,839 under graduate regular students in its five faculties and four schools comprising of 32 departments. One RH and Anti HIVAIDS Club was operating in the university.

At the beginning, students were stratified based on their year of study, as first, second, and third year and above. From the five faculties, students in each batch were selected using the simple random sampling technique. The number of students sampled from each faculty was proportional to the total number of students thereof. Regarding knowledge of reproductive and sexual rights, students who scored above the mean score in the knowledge questions were considered knowledgeable. Data were collected using a pretested structured self-administered questionnaire. Twelve data collectors guided by one supervisor collected the data on April 7, 2012. The study participants filled the questionnaire at the same time in 12 lecture halls, each with 200 seats. Only 55 students sat in each lecture hall. Female and male students were placed in different rooms to ensure their privacy. Data quality was controlled by giving trainings and appropriate supervisions for data collectors. The overall supervision was carried out by the principal investigator. A pre-test was conducted on 5% of the questionnaire on students of Gondar University. Appropriate modifications were made after analyzing the pre-test result before the actual data collection.

The questionnaires filled by the students were checked for completeness and entered into EPI INFO version 3.5.3 statistical software and then exported to SPSS version 20 for further analysis. Descriptive statistics was used to describe the study population in relation to relevant variables. Both bivariate and multivariate logistic regression models were used to identify associated factors. Odds Ratios and their 95% Confidence Intervals were computed and variables with p - value less than 0.05 were considered as significantly associated with the outcome variable.

Ethical clearance was obtained from the Institute of Public Health, the University of Gondar. A formal letter of cooperation was written to Wolaita Sodo University. Verbal consent was obtained from each study participant.

## Results

### Socio-demographic characteristics of the study participants

Out of the expected 660 respondents, 648 agreed to participate in the study, yielding a response rate of 98.1%. Out of this, six incomplete questionnaires were discarded. The age of the respondents ranged from 18 to 28, with the median age of 20 years. More than half of the respondents (57.4%) were between the ages of 18 and 20 years. Male respondents were 516 (80.4%). Two hundred eighty (43.6%) of the respondents were Orthodox Christians, while 154 (24%) of the study participants were Wolaita by ethnicity. Regarding marital status, almost all (95.5%) of the study participants were single, and around half (52%) were from rural areas (Table 1).

**Table 1:** Socio-demographic and academic characteristics of Wolaita Sodo University undergraduate students, 2012, N = 642

**Variables**	**Frequency**	**Percentage %**
**Age**		
**Median 20.00, Range 18–25**		
**Sex**		
**Male**	516	80.4
**Female**	126	19.6
**Religion**		
**Orthodox**	280	43.6
**Protestant**	233	36.3
**Muslim**	109	17
**Others**	20	3.1
**Place where they came from**		
**Rural**	334	52
**Urban**	308	48
**Ethnic origin**		
**Wolaita**	154	24
**Amhara**	141	22
**Oromo**	100	15.6
**Hadiya**	55	8.6
**Tigre**	43	6.6
**Others**	149	23.2
**Type of school attended (elementary and high school)**		
**Governmental**	472	73.5
**Nongovernmental**	109	17
**Both**	61	9.5
**Faculty**		
**Engineering**	161	25.1
**Natural & computational sciences**	156	24.5
**Business and economics**	94	14.6
**Social science and humanities**	94	14.6
**Agriculture**	67	10.4
**Health sciences**	42	6.5
**Law**	20	3.1
**Veterinary medicine**	8	1.2
**Year of study**		
**Year I**	336	52.3
**Year II**	180	28
**Year III+**	126	19.7

### Sexual experience and RH service utilization

Two hundred six (32.1%) of the respondents reported that they had sexual experience. The mean age at the first sex was 17.34. Out of those who had sexual experience, 158 (53.3%) said that they had multiple sexual partners in their life time. One hundred six (16.5%) of the respondents reported that it was not important to discuss sexual issues with parents, while 140 (21.8%) had not discussed about the issue with anyone else. Among those who had discussed sexual issues, 419 (65.3%) discussed with their friends. Only 229 (35.7%) of the respondents participated in RH clubs.

Around one-third of the respondents (37.1%) had no awareness that the student clinic in the campus provides RH services, while only 156 (24.3%) ever used any of the RH services.

Around half 48.8% of the respondents said that their sources of information regarding reproductive and sexual issues were their peers, while health personnel, the media, teachers and parents were sources of information to 45.2%, 43.5%, 31% and 28.2%, respectively.

### Knowledge of reproductive and sexual rights

Participants were asked 24 questions to assess their knowledge on reproductive and sexual rights, and they were categorized in to two groups based on their score in relation to the mean. The mean score was 15.7. More than half (54.5%) of the respondents were found to be knowledgeable, while a substantial proportion (45.5%) of the respondents was not.

Students were asked whether a married woman should have the right to limit the number of her children according to her desire and without her husband’s consent. The majority (63.7%) of them showed their disagreement with this idea. One hundred fifty-seven (24.5%) of the study participants said that a husband should get sex whenever he wants irrespective of his wife’s wish. Around half (53.7%) disagreed with the question that reflected the right of girls to autonomous reproductive choices without their partners` consent. Four hundred nine (63.7%) agreed that parents have the right to decide on sexual and RH issues of their children. Among all, 270 (42.1%) of the respondents disagreed with the statement which said students should have the right to freedom of assembly and political participation to influence the Government to place sexual and reproductive health issues on the priority list during planning and interventions. Three hundred sixty-four (56.7%) agreed with the statement that unmarried couples have no right to use contraceptives other than condoms (Table 2).

**Table 2:** Rresponses of the study participants to the knowledge questions, wolaita Sodo University, Wolaita Sodo, SNNPRS, Ethiopia, 2012, N = 642

**Questions**	**Yes**		**No**	
	**Freq.**	**%**	**Freq.**	**%**
Do youths have the right that their use of reproductive health services is kept confidential?	500	77.9	142	22.1
A man should get sex whenever he wants irrespective to his wife’s wish.	157	24.5	485	75.5
Does a married woman have the right to limit the number of her children according to her desire without her husband’s consent?	233	36.3	409	63.7
Husband has no obligation to share childcare?	155	24.1	487	75.9
Do girls have the right to resist genital mutilation against their families will?	501	78	141	22
Do youths have a full right to access all RHSs without parents’ consent?	450	70.1	192	29.9
Do girls have the right to autonomous reproductive choices without their partners consent?	297	46.3	345	53.7
Do you think that all students must be free to enjoy and control their sexual and reproductive life?	380	59.2	262	40.8
Do unmarried woman have the right to maternity leave with adequate social security benefits?	391	60.9	251	39.1
Unmarried couples have no right to use contraceptives other than condoms.	364	56.7	278	43.3

### Factors associated with knowledge of sexual and reproductive rights

The type elementary and high school attended, faculty, place they came from, participation in RH clubs, discussion RH issues, and utilization of RH services were found to have significant and independent effect on knowledge of reproductive and sexual rights, while sexual experience, parental education and occupation were not significantly associated.

Students who attended private elementary and high schools were about 2 times more likely to be knowledgeable than students from public schools [AOR: 2.08, 95% CI: 1.08, 3.99]. Respondents who came from urban areas were also about 1.5 times more likely to be knowledgeable compared to those who came from rural areas [AOR: 1.45, 95% CI: 1.01, 2.11]. Study participants from faculty of health sciences were about 3 times more likely to be knowledgeable than students of social science and humanities [AOR: 2.98, 95% CI: 1.22, 7.30].

Those who participate in RH clubs were about 3 times more likely to be knowledgeable than those who did not participate [AOR: 3.11, 95% CI: 2.08, 4.65]. Students who utilized RH services were 2 times more likely to be knowledgeable than those who did not use [AOR: 2.34, 95% CI: 1.5, 3.69]. Students who ever had discussed RH issues with someone else were 2 times more likely to be knowledgeable than those who did not [AOR: 2.31, 95%CI: 1.48, 3.62] (Table 3).

**Table 3:** Bivariate and multivariate logistic regression analysis output of factors associated with knowledge of RSR among WSU students, SNNPRS Ethiopia, 2012

**Variables**		**Knowledge**		**Crude OR [95% CI]**	**Adjusted OR [95% CI]**
		**Knowledgeable**	**Not knowledgeable**		
**Place where they came from**	rural	162 (46.3%)	174 (59.6%)	1	1
	urban	188 (53.7%)	118 (40.4%)	1.71[1.25,2.34]	1.45[1.01, 2.11]*
**Type of school attended**	Public	232 (79.5%)	240 (68.6%)	1	1
	Private	68 (19.4%)	41 (14.0%)	1.60[1.04, 2.45]	2.08[1.08, 4.00]*
	Both	42 (12.0%)	19 (6.5%)	2.13[1.207,3.78]	1.18[.719, 1.95]*
**Faculties**	Engineering	88 (25.1%)	73 (25.0%)	.974[.584, 1.62]	.822[.465,1.45]*
	NCS	91 (26.0%)	65 (22.3%)	1.13[.675, 1.89]	1.201[.68**,** 2.12]
	Agriculture	29 (8.3%)	38 (13.0%)	.616[.328, 1.15]	.691[.343, 1.39]
	Veterinary	2 (0.6%)	6 (2.1%)	.269[.052, 1.40]	.398[.073, 2.18]
	FBE	43 (12.3%)	51 (17.5%)	.681[.383, 1.21]	.545[.285, 1.04]
	Health	33 (9.4%)	9 (3.1%)	2.96[1.27,6.87]	2.98[1.22, 7.30]*
	Law	12 (3.4%)	8 (2.7%)	1.21[.453, 3.23]	.884[.298, 2.62]
	SSH	52 (14.9%)	42 (14.4%)	1	1
**Participate in RH clubs**	Yes	174 (49.7%)	55 (18.8%)	4.26[2.97, 6.10]	3.11[2.08,4.65]*
	No	176 (50.3%)	237 (81.2%)	1	1
**Important to discuss sexual issues**	Yes	321 (91.7%)	215 (73.6%)	3.96[2.50,6.28]	
	No	29 (8.3%)	77 (26.4%)	1	1
**Had discussed RH issues**	Yes	308 (88.0%)	194 (66.4%)	3.70[2.474,5.54]	**2.31[1.48, 3.61]**
	No	42 (12.0%)	98 (33.6%)	1	1
**Ever used RH services**	Yes	117 (33.4%)	39 (13.4%)	3.25[2.17,4.87]	2.34[1.48,3.69]*
	No	233 (66.6%)	253 (86.6%)	1	1
**Paternal education**	No education	99 (28.3%)	119 (40.8%)	1	1
	Elementary school	125 (35.7%)	102 (34.9%)	1.47[1.01, 1.01]	
	Secondary school	63 (18.0%)	39 (13.4%)	1.94[1.20, 3.13]	
	College +	63 (18.0%)	32 (11.0%)	2.36[1.43, 3.91]	
**Maternal education**	No education	153 (43.7%)	154 (52.7%)	1	1
	Elementary school	121 (34.6%)	91 (31.2%)	1.33[.94, 1.90]	
	Secondary school	47 (13.4%)	36 (12.3%)	1.31[.80, 2.14]	
	College +	29 (8.3%)	11 (3.8%)	2.65[1.28, 5.50]	
**Maternal occupation**	Housewife	171 (48.9%)	164 (56.2%)	1	1
	Government employee	30 (8.6%)	13 (4.5%)	2.87[.29, 27.94]	
	Private employee	23 (6.6%)	15 (5.1%)	2.21[1.11, 4.39]	
	Trader	59 (16.9%)	35 (12.0%)	1.47[.74,2.91]	
	Farmer	64 (18.3%)	64 (21.9%)	1.61[1.01, 2.58]	
	Others	3 (0.9%)	1 (0.3%)	.959[.63, 1.44]	

## Discussion

Reproductive health targeted Millennium Development Goals (MDGs) will not be achieved without improving access to reproductive health services [5]. But the youth are experiencing all forms of sexual abuse and sexual-rights violations that affect their lives [14] and the majority of the young people have very little knowledge of what sexual rights they are entitled to. Sometimes, they do not even appreciate the extent of their violations, and what is worse still, they do not know where they could go for legal or social advice [8]. Thus, this study is aimed to assess the knowledge of reproductive and sexual rights and associated factors among Wolaita Sodo University students.

In this study, a substantial proportion of students were not knowledgeable. As university students are the educated segment of the population, are expected to be the next leaders of the nation, this level of knowledge is far below adequate.

Around two third of the respondents did not accept that a married woman has the right to limit the number of her children according to her desire without her husband’s consent. Furthermore a large group did not know that a married woman has the right to say no to sex, regardless of her husband`s wishes. This finding was lower than that of a study conducted in US, Texas [15]. This disparity might be because of the difference in culture and norm in which the two populations were brought up. Sex and sexuality are taboo in most Ethiopian cultures, resulting in reluctance to discuss and address sexual health issues [16]. Furthermore, the Ethiopian society is highly patriarchal where women’s right is undermined [17]. For most African societies, lack of knowledge on sexual matters entails safety as it is assumed that if adolescents are not exposed to such knowledge, the likelihood of getting involved, and consequently becoming victims, will be slim [18].

Among the respondents, about one-fifth of the females and one-fourth of males agreed that a husband should get sex whenever he wants irrespective of his wife’s wish. This finding is better than a similar study conducted among Indian adolescents selected from five states representing different cultural settings, where above 40% of women agreed to it [19]. This difference might be attributed to the difference in age and educational level of the study participants.

Students who came from urban areas were more likely to be knowledgeable compared to those who came from rural areas. The possible explanation for this scenario can be that students from towns relatively have better access to information through youth associations, youth centers, the media and the environment itself because most of NGO services are limited to urban areas. However, their counterparts from rural areas might lack such chances because of low awareness of the society which inhibits free and open discussion about reproductive and sexual issues.

Students who had attended private elementary and high schools were more likely to be knowledgeable than students from public schools. This finding is in contrary to the Nigerian study where students attending public schools were more aware of RSR than students from private schools [20]. In Nigeria, NGOs working on RH give more emphasis to public schools than to the private schools. But in Ethiopia, the number of NGOs working in public elementary and high schools is low. Additionally, compared to public schools, most of the private schools are found in towns where the students are more familiar with this issue and have better access to youth centers and sexuality education. Anti HIV/AIDS and RH clubs are also relatively strong and functional in private schools. Besides, students of private schools are relatively from families with good educational and economic background compared to students from public schools.

Health sciences students were more likely to be knowledgeable than students in the faculty of Social Sciences and Humanities (SSH). This might be due to courses that health science students are taking, such as RH which includes reproductive and sexual rights as a chapter. In addition, they can learn this specific topic in one or another way at least because their instructors are well familiarized and even experts on RH issues. Reproductive health service utilization has impact on the knowledge of reproductive and sexual rights. Students who utilized RH services were more likely to be knowledgeable than those who did not. This finding is in agreement with US study where users of RH services had more inclination towards reproductive health rights, and inconsistent contraceptive use was associated with low sexual assertiveness [15]. The possible explanation can be that since the services are provided by expertise that can give clear and correct information and answer the questions of their clients, utilization of RH services increases the probability of getting counseling and accurate information which has a direct impact on the knowledge of sexual and reproductive rights.

Discussing reproductive and sexual issues affects the knowledge of reproductive and sexual rights in a positive way. Students who have ever had discussed RH issues were more likely to be knowledgeable than those who did not. This can be explained by the fact that knowledge gained through experience sharing during discussion can increase the knowledge of reproductive and sexual rights. This study has shared the limitations of cross-sectional studies i.e. the difficulty of determining causal relationships between variables. As a cross-sectional study requires respondents to remember information retrospectively, recall bias are the other potential limitations of this study. However, scientific procedures were employed to minimize possible effects. In addition, supervision, pretest of the data collection tool, and adequate training of data collectors and supervisors were utilized.

## Conclusions

The level of knowledge of the respondents is found to be inadequate. Attending private elementary and high schools, coming from urban areas, being second and third year student, belonging to the faculty of health sciences, discussing reproductive and sexual issues, participating in RH clubs, and utilization of RH services showed a positive and significant association with knowledge of reproductive and sexual rights. The Ministry of Education of Ethiopia has to incorporate reproductive and sexual rights in the curricula of high schools and institutions of higher learning.

## Competing interests

The authors declare that they have no competing interests.

## Authors' contributions

YM wrote the proposal, participated in data collection, analyzed the data and drafted the paper. AB and ZB approved the proposal with some revisions, participated in data analysis and revised subsequent drafts of the paper. All authors read and approved the final manuscript.

## Pre-publication history

The pre-publication history for this paper can be accessed here:

http://www.biomedcentral.com/1472-698X/13/12/prepub
